# Gut Microbiota-Brain Axis in Pancreatic Cystic Neoplasms: An Observational Analysis

**DOI:** 10.12669/pjms.41.12.12010

**Published:** 2025-12

**Authors:** Ibrahim Halil Ocal, Omer Basol, Abdullah Oguz, Huseyin Bilge

**Affiliations:** 1 Ibrahim Halil Ocal, Department of General Surgery, Adıyaman Training and Research Hospital, Adıyaman, Turkey; 2 Omer Basol, Department of General Surgery, University of Health Sciences, Diyarbakır Gazi Yaşargil Health Application and Research Centre, Diyarbakır, Turkey; 3 Abdullah Oguz, Department of General Surgery, Dicle University, Diyarbakır, Turkey; 4 Huseyin Bilge, Department of General Surgery, University of Health Sciences, Diyarbakır Gazi Yaşargil Health Application and Research Centre, Diyarbakır, Turkey

**Keywords:** Brain-Gut Axis, Gastrointestinal Microbiome, Pancreatic Cystic Neoplasms

## Abstract

**Objective:**

This observational analysis aimed to explore the association between gut microbiota and brain axis in pancreatic cysts and assess the impact of this association on clinical outcomes.

**Methodology:**

This retrospective cohort study was conducted at Dicle University Faculty of Medicine, Diyarbakır, Turkey. Forty-seven patients (serous cystadenoma n=16, mucinous neoplasm n=31) treated at a single center between 2015 to 2023 were included in the study. Microbiota analysis (16S rRNA sequencing) of stool samples, biochemical and hormonal parameters from blood samples were evaluated. Depression-anxiety scales and cognitive tests were also performed.

**Results:**

Microbiota diversity in the mucinous neoplasm group (Shannon index: 2.9±0.6) was significantly lower than in the serous cystadenoma group (3.8±0.5) (p=0.012). Firmicutes/Bacteroidetes ratio (2.89±0.5), inflammatory markers (CRP: 4.2±4.8 mg/dL) and depression scores (Beck: 18.6±6.4) were significantly higher in mucinous neoplasm patients (p<0.05). Two-year overall survival rates were 100% in the serous cystadenoma group and 80% in the mucinous neoplasm group (p=0.015).

**Conclusion:**

The microbiota-brain axis has an important role in pancreatic cysts. Microbiota imbalance, increased inflammation and high depression-anxiety levels observed especially in mucinous neoplasms suggest potential targets for future therapeutic interventions in this patient group.

## INTRODUCTION

Pancreatic cystic lesions are diagnosed more frequently in recent years due to advances in imaging techniques. These lesions may be benign, precancerous or malignant. Particularly, cysts located in the pancreas with neuroendocrine differentiation represent a diagnostic and management challenge to the clinician. These cysts are derived from endocrine cells of the pancreas.^1^ Recent research indicates that, apart from gastrointestinal disorders, microbiota also influences the development of neurological and psychiatric conditions.^2^ The interaction between the microbiota and the central nervous system is known as the microbiota-brain axis, and it occurs via the neurotransmitters, immune system, and endocrine system. This axis is thought to play a role in the pathogenesis and clinical course of pancreatic cysts.^3^

The management of pancreatic cystic lesions varies according to the type, size and malignancy potential of the lesion. In particular, mucinous cystic neoplasms (MCN) and intraductal papillary mucinous neoplasms (IPMN) are considered to be lesions at risk of malignancy, whereas serous cystadenomas (SCN) are generally considered benign.^4^ However, the correct identification and management of these lesions is still a significant challenge in clinical practice.^5^

Conditions affecting the pancreas, like chronic pancreatitis, profoundly lower the quality of life while also presenting a systemic inflammatory component. This inflammation can also affect the neuropsychological status of patients.^6^ Furthermore, pancreatic cystic tumours originate from the islands of Langerhans of the pancreas and have highly variable biological behaviour. Cystic forms of these tumours have been diagnosed more frequently in recent years and their biological behaviour is still a matter of debate.^7^

Research findings show that probiotic supplements help control gut bacteria while reducing body-wide inflammation according to recent scientific studies if idobacteria and Lactobacilli were added to their conventional therapy because these probiotics. The research by Liu et al demonstrated that patients with chronic inflammatory conditions received better treatment results when B restored intestinal microbiota balance and decreased inflammatory markers. The dual therapy showed promise for treating different gastrointestinal and systemic diseases because it successfully managed the equilibrium between anti-inflammatory and pro-inflammatory cytokines.^8^ In the current literature, there is limited information on the relationship between pancreatic cysts and the microbiota-brain axis. In particular, it is not fully understood how the microbiota profiles of such cysts change and how these changes are reflected in clinical outcomes.^9^ Furthermore, the role of the microbiota-brain axis in the pathogenesis of pancreatic cysts and the effect of this relationship on the neuropsychological status of patients is not yet clear.^2^

It is known that the current criteria for the diagnosis and management of pancreatic cystic lesions are inadequate. Especially, diagnostic approaches to distinguish between mucinous and non-mucinous or neoplastic and non-neoplastic benign cysts appear to be unsatisfactory.^10^ Therefore, this observational study investigates the microbiota-brain axis in patients with pancreatic cysts and how this association may affect clinical outcomes. This aims to provide insights for developing innovative strategies in the diagnosis and characterization of pancreatic cysts.

## METHODOLOGY

This single-centre retrospective cohort study was conducted at Dicle University Faculty of Medicine, Department of General Surgery, Diyarbakır, Turkey. The data of the patients included in the study were retrospectively analysed from hospital records between 2015 and 2023. The study protocol was evaluated and approved by the Dicle University Faculty of Medicine Non-Interventional Clinical Research Ethics Committee (Meeting Number: 198, Date: March 25, 2021). The study protocol was conducted in accordance with the principles of the Declaration of Helsinki.

### Study population:

Analysis of hospital records identified 47 patients who were followed up with the diagnosis of pancreatic cyst and underwent surgical treatment at our institution between 2015 and 2023. The cohort consisted of 16 patients diagnosed with serous cystadenoma and 31 with mucinous neoplasm.

### Inclusion and Exclusion Criteria:

Inclusion criteria comprised patients over 18 years of age who had undergone surgical treatment with routine postoperative follow-up. The study excluded patients who had undergone pancreatic surgery before or had active infections or immunosuppressive conditions or received chemotherapy or radiotherapy before surgery or failed microbiota analysis. The researchers conducted patient selection according to systematic procedures to achieve reliable data. Each patient was analyzed against the study criteria for selection and only then was included in the study group.

The data used in the study were obtained from hospital registration systems, pathology reports and laboratory results. During data collection, care was taken for patient confidentiality and all data were anonymised and recorded. Demographic data, clinical findings, tumour localisation, surgical methods, laboratory parameters, inflammatory markers, microbiota composition and psychological evaluation results were analysed.

Clinical data such as age, gender, tumour localisation, pathological diagnosis, surgical method, operation time and hospital stay were recorded. Tumour localisations were divided into three groups as pancreatic head, corpus and tail. The operation types were divided into two main categories as distal pancreatectomy and Whipple procedure. The duration of hospitalisation and operation time were used to evaluate the postoperative period. All surgical procedures were performed by the same surgical team.

### Microbiota analysis:

Microbiota analysis is not routinely performed for pancreatic cyst patients at our institution. This analysis was conducted specifically for research purposes in this study. For microbiota analysis, stool samples were collected from all participating patients in the preoperative period within 24-48 hours before surgery, and microbial profiling was performed using 16S rRNA sequencing method. Shannon index and Chao1 index were calculated as diversity and richness indices. Dominant bacterial species were evaluated based on the ratio of Firmicutes and Bacteroidetes. The abundance ratios of Lactobacillus spp., Bifidobacterium spp. and Clostridium spp. were analyzed. In addition, the presence of pathogenic bacteria was evaluated statistically and compared between the groups.

Biochemical profiles of the patients were analyzed on blood samples. Inflammation level was evaluated by measuring CRP, IL-6 and TNF-α levels among inflammatory markers. Serotonin and chromogranin A levels were analyzed among neuroendocrine markers. Fasting glucose, insulin and HbA1c levels were evaluated among metabolic parameters, and CEA and CA 19-9 levels were analyzed among tumour markers.

Psychological evaluation is not part of the routine clinical assessment for pancreatic cyst patients at our center. These assessments were performed specifically for this research study. Beck Depression Inventory, GAD-7 Anxiety Scale and Hamilton Anxiety Scale were used to measure depression and anxiety levels. Cognitive functions were measured by Mini Mental State Examination (MMSE), Montreal Cognitive Assessment Test (MoCA) and Clock Drawing Test. In addition, dopamine, noradrenaline and GABA levels were analyzed in terms of neurotransmitter levels.

The surgical methods, postoperative complications, presence of lymphovascular invasion, adjuvant treatment, disease recurrence and survival times were recorded in detail. Perioperative complications were evaluated in terms of bleeding, infection and fistula development. Disease recurrence was determined according to clinical and radiological follow-up findings. Survival analyses included two-year overall and disease-free survival rates.

### Statistical analysis:

All data were analyzed using SPSS (Statistical Package for the Social Sciences) 26.0 software. Chi-square test was used for the comparison of categorical variables, Student’s t-test was used for data conforming to parametric distribution and Mann-Whitney U test was used for data not conforming to normal distribution in the analysis of continuous variables. Spearman’s method was preferred for correlation analyses. In multivariate analyses, logistic regression analysis was applied to evaluate the relationship between independent variables and disease progression. In addition, linear regression analysis was used for the factors affecting the microbiota-brain axis relationship. Survival analyses were calculated by Kaplan-Meier method and differences between groups were compared by Log-rank test.

## RESULTS

The research included 47 patients with pancreatic cystic neoplasms who had 16 serous cystadenomas (34.0%) and 31 mucinous neoplasms (66.0%). The patient population consisted mainly of females (n=38, 80.9%) with an average age of 54.9±16.0 years. The pancreatic tail proved to be the most frequent location for tumours (n=25, 53.2%) while the head and body of the pancreas received fewer occurrences (n=19, 40.4% and n=3, 6.4% respectively). The surgical procedures performed included Whipple procedure (n=25, 53.2%) and distal pancreatectomy (n=22, 46.8%) with patients undergoing operations lasting 378.0±172.4 minutes and spending 22.0±8.5 days in the hospital. The preoperative laboratory results indicated different levels of inflammation through albumin (3.6±0.5 g/dL) and hemoglobin (13.2±1.4 g/dL) and lactate dehydrogenase (191.7±38.2 U/L) and C-reactive protein (3.2±4.1 mg/dL) measurements (Table-I).

**Table-I:** Demographic and Clinical Characteristics

Characteristic	Value	p-value
Number of Patients (n)	47	—
Age (years) (Mean ± SD)	54.9 ± 16.0	—
***Gender, n (%)***		0.002*
- Female	38 (80.9%)	
- Male	9 (19.1%)	
***Tumor Location, n (%)***		0.041*
- Pancreatic Head	19 (40.4%)	
- Pancreatic Body	3 (6.4%)	
- Pancreatic Tail	25 (53.2%)	
***Pathological Diagnosis, n (%)***		0.315
- Serous Cystadenoma	16 (34.0%)	
- Mucinous Neoplasm	31 (66.0%)	
***Type of Surgery, n (%)***		0.124
- Distal Pancreatectomy	22 (46.8%)	
- Whipple Procedure	25 (53.2%)	
Length of Hospital Stay (days) (Mean ± SD)	22.0 ± 8.5	—
Duration of Surgery (minutes) (Mean ± SD)	378.0 ± 172.4	—
***Preoperative Laboratory Parameters (Mean ± SD)***		
- Albumin (g/dL)	3.6 ± 0.5	—
- Lactate Dehydrogenase (LDH) (U/L)	191.7 ± 38.2	—
- C-reactive Protein (CRP) (mg/dL)	3.2 ± 4.1	—
- Hemoglobin (g/dL)	13.2 ± 1.4	—

***Notes:*** Data are presented as mean ± standard deviation (SD) or n (%). The chi-square test was used for categorical variables, while the Student’s t-test was used for continuous variables. The p-values indicate comparisons between groups.

* p < 0.05 was considered statistically significant. ***Abbreviations:*** SD: standard deviation; LDH: lactate dehydrogenase; CRP: C-reactive protein.

The serous cystadenoma group showed greater bacterial diversity than the mucinous neoplasm group according to microbiota diversity analysis. Shannon and Chao1 diversity indices were found to be statistically significantly higher in the serous cystadenoma group. When the distribution of bacterial groups was analysed, it was observed that the proportion of Firmicutes was significantly higher in the mucinous neoplasm group. On the other hand, Bacteroidetes ratio was found to be lower in this group. Especially the higher Firmicutes/Bacteroidetes ratio in the mucinous neoplasm group suggests that the microbial balance has changed and this change may have an effect on the disease process. When specific bacterial species were analysed, it was determined that Lactobacillus and Bifidobacterium species were more predominant in the serous cystadenoma group, whereas Clostridium species were more predominant in the mucinous neoplasm group. In addition, the presence of pathogenic bacteria was significantly higher in the mucinous neoplasm group (Table-II).

**Table-II:** Gut Microbiota Composition Analysis.

Parameter	Serous Cystadenoma (n=16)	Mucinous Neoplasm (n=31)	p-value
***Alpha Diversity Indices***			
Shannon Index	3.8 ± 0.5	2.9 ± 0.6	0.012*
Chao1 Index	245.3 ± 34.2	198.6 ± 28.7	0.025*
***Phylum-Level Composition (%)***			
Firmicutes	52.3 ± 8.4	64.7 ± 9.2	0.018*
Bacteroidetes	31.5 ± 6.2	22.4 ± 5.8	0.022*
F/B Ratio	1.66 ± 0.3	2.89 ± 0.5	0.008*
***Genus-Level Abundance (%)***			
Lactobacillus spp.	12.4 ± 3.1	8.2 ± 2.8	0.031*
Bifidobacterium spp.	8.6 ± 2.4	5.3 ± 1.9	0.028*
Clostridium spp.	15.2 ± 4.2	23.8 ± 5.1	0.015*
***Pathogenic Bacteria, n (%)***			0.042*
Present	3 (18.8)	22 (71.0)	
Absent	13 (81.2)	9 (29.0)	

***Note:*** All values presented as mean ± SD or n (%).

* p < 0.05 considered statistically significant. ***Abbreviations:*** F/B, Firmicutes/Bacteroidetes.

The biochemical and hormonal tests showed that mucinous neoplasm patients had higher levels of inflammatory markers. CRP and IL-6 levels among inflammatory markers were significantly higher in the mucinous neoplasm group. Similarly, TNF-α level was higher in the mucinous neoplasm group and it is thought that this group may be more related with inflammatory processes. In terms of metabolic parameters, fasting glucose and insulin levels were higher in mucinous neoplasm patients compared to serous cystadenoma group. HbA1c values were also higher in the mucinous neoplasm group, indicating that these patients may have a higher metabolic risk. In terms of tumour markers, CA 19-9 and CEA levels were significantly higher in the mucinous neoplasm group (Table-III).

**Table-III:** Biochemical, Inflammatory, and Neuroendocrine Markers

Parameter	Reference Range	Serous Cystadenoma (n=16)	Mucinous Neoplasm (n=31)	p-value
***Inflammatory Markers***				
CRP (mg/dL)	0–5	1.8 ± 1.6	4.2 ± 4.8	0.042*
IL-6 (pg/mL)	0–7	3.2 ± 1.4	7.8 ± 2.6	0.015*
TNF-α (pg/mL)	0–20	12.4 ± 4.2	18.6 ± 5.8	0.028*
***Neuroendocrine Markers***				
Serotonin (ng/mL)	50–200	125.4 ± 45.2	168.7 ± 52.3	0.122
Chromogranin A (ng/mL)	25–140	68.5 ± 24.6	95.8 ± 32.4	0.084
***Metabolic Parameters***				
Fasting Glucose (mg/dL)	70–100	98.2 ± 12.4	112.6 ± 18.5	0.045*
Insulin (μU/mL)	2.6–24.9	8.4 ± 3.2	12.8 ± 4.6	0.038*
HbA1c (%)	4–6	5.6 ± 0.4	6.2 ± 0.6	0.032*
***Tumor Markers***				
CEA (ng/mL)	0–5	2.8 ± 1.2	4.6 ± 2.1	0.046*
CA 19-9 (U/mL)	0–37	22.4 ± 8.6	42.8 ± 15.4	0.012*

***Note:*** All values mean ± SD. *p < 0.05 considered statistically significant. ***Abbreviations:*** CRP, C-reactive protein; IL-6, interleukin-6; TNF-α, tumor necrosis factor-alpha; HbA1c, hemoglobin A1c; CEA, carcinoembryonic antigen; CA 19-9, cancer antigen 19-9.

The results of neuropsychological assessment showed that patients with mucinous neoplasms had significantly higher depression and anxiety scores than those with serous cystadenomas. Beck Depression Inventory, GAD-7 Anxiety Score and Hamilton Anxiety Scale results were significantly higher in the mucinous neoplasm group. However, in terms of cognitive function tests, MMSE and MoCA scores were within normal limits in both groups. In terms of neurotransmitter levels, no significant difference was found between the groups in dopamine, noradrenaline and GABA levels (Table-IV).

**Table-IV:** Neuropsychological and Cognitive Assessment

Assessment Tool	Score Range/Criteria	Serous Cystadenoma (n=16)	Mucinous Neoplasm (n=31)	p-value
Depression & Anxiety Scales				
Beck Depression Inventory	0–63†	14.2 ± 5.8	18.6 ± 6.4	0.032*
GAD-7	0–21‡	8.4 ± 3.2	11.8 ± 4.1	0.028*
Hamilton Anxiety Scale	0–56	12.6 ± 4.8	16.4 ± 5.2	0.042*
Cognitive Function				
MMSE	0–30¦	27.8 ± 1.6	26.9 ± 1.8	0.324
MoCA	0–30¶	25.6 ± 2.1	24.8 ± 2.4	0.456
Clock Drawing Test	0–5#	4.2 ± 0.6	4.0 ± 0.8	0.582
Neurotransmitters				
Dopamine (pg/mL)	—	42.6 ± 12.4	38.4 ± 11.8	0.248
Noradrenaline (pg/mL)	—	328.4 ± 85.6	342.6 ± 92.4	0.754
GABA (μmol/L)	—	2.8 ± 0.6	2.6 ± 0.8	0.684

***Scoring Criteria:*** †Beck: 0–9 minimal, 10–16 mild, 17–29 moderate, 30–63 severe depression. ‡GAD-7: 0-4 minimal, 5–9 mild, 10–14 moderate, 15–21 severe anxiety. ¦MMSE: 24–30 normal, 18–23 mild impairment, <18 severe impairment. ¶MoCA: ≥26 normal, <26 cognitive impairment, #Clock: 5 normal, <3 impairment. ***Abbreviations:*** GAD-7, Generalized Anxiety Disorder-7; MMSE, Mini-Mental State Examination; MoCA, Montreal Cognitive Assessment; GABA, gamma-aminobutyric acid.

* p < 0.05 considered statistically significant.

### Surgical outcomes and follow-up:

The analysis of surgical results showed different patterns between the two groups. The surgical approach for serous cystadenomas involved distal pancreatectomy in 81.3% of cases but mucinous neoplasms needed Whipple procedure in 71.0% of cases (p=0.024). The longer surgical duration in mucinous neoplasms (432.5±186.8 vs 285.0±95.2 minutes, p=0.018) resulted from tumor placement rather than the specific histological characteristics. The study results showed that pancreatic head tumors needed Whipple procedure and resulted in longer surgery times than tail tumors that underwent distal pancreatectomy. The main factor that extended surgical time was the location of the tumor because pancreaticoduodenectomy procedures take longer than distal resections due to their complex anatomy. The hospital stay duration for mucinous neoplasms exceeded that of serous cystadenomas by 5.6 days (24.2±9.2 vs 18.6±6.4 days, p=0.035) while complications occurred more frequently in mucinous neoplasms (71.0% vs 18.7%, p=0.042).

The pathological study showed that lymphovascular invasion occurred only in mucinous neoplasms at a rate of 38.7% compared to 0% in other types (p=0.038). The need for adjuvant treatment was significantly higher in the mucinous neoplasm group and the need for additional oncological treatment after surgery was found to be higher in these patients. The recurrence rate was also higher in the mucinous neoplasm group and the risk of recurrence was higher in this group. When the survival analyses were examined, it was determined that the two-year overall survival rates were higher in the serous cystadenoma group (100% vs 80%, p=0.015), with disease-free survival rates of 100% versus 70%, respectively (p=0.018) (Table-V).

**Table-V:** Surgical Outcomes and Follow-up Data.

Parameter	Serous Cystadenoma (n=16)	Mucinous Neoplasm (n=31)	p-value
***Surgical Details***			
Procedure Type, n (%)			0.024*
- Distal Pancreatectomy	13 (81.3)	9 (29.0)	
- Whipple Procedure	3 (18.7)	22 (71.0)	
***Operation Duration (min)***	285.0 ± 95.2	432.5 ± 186.8	0.018*
Hospital Stay (days)	18.6 ± 6.4	24.2 ± 9.2	0.035*
***Pathological Findings***			
Lymphovascular Invasion, n (%)	0 (0.0)	12 (38.7)	0.038*
***Postoperative Course***			
Perioperative Complications†, n (%)	3 (18.7)	22 (71.0)	0.042*
Adjuvant Therapy, n (%)	0 (0.0)	16 (51.6)	0.028*
***Long-term Outcomes***			
Follow-up Duration (months)	24.5 ± 8.2	22.8 ± 7.6	0.648
Disease Recurrence, n (%)	0 (0.0)	9 (29.0)	0.032*
***2-Year Survival Rates (%)***			
Overall Survival	100.0	80.0	0.015*
Disease-Free Survival	100.0	70.0	0.018*

†Complications include bleeding, fistula formation, and surgical site infection.

### Statistical methods:

Chi-square test for categorical variables; Student’s t-test for continuous variables; Kaplan-Meier method for survival analysis with log-rank test for group comparisons. *p < 0.05 considered statistically significant.

**Fig.1:**
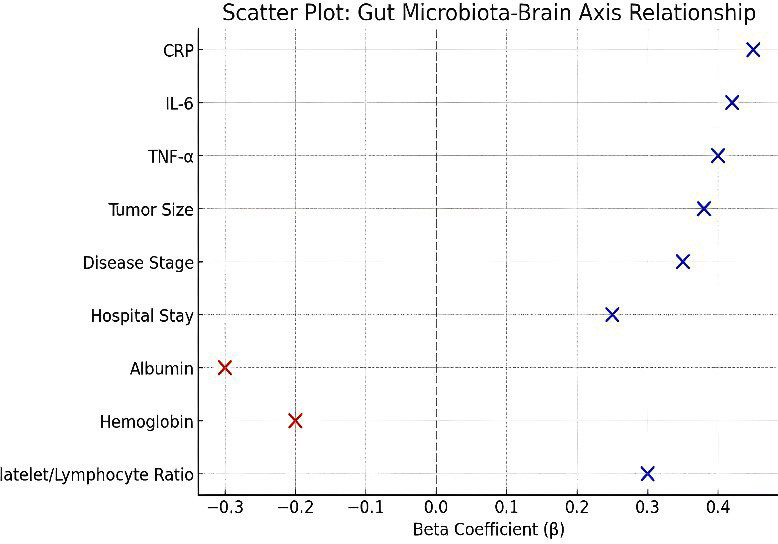
Forest Plot of Gut Microbiota-Brain Axis Correlations. Forest plot illustrating correlation coefficients (r) and 95% confidence intervals between gut microbiota diversity indices, bacterial taxa, inflammatory markers, and neuropsychological parameters. Negative correlations extend leftward, positive correlations rightward from the null effect line (r = 0).

The microbiota diversity linked to reduced depression and anxiety symptoms. The Firmicutes/Bacteroidetes ratio produced negative associations with serum serotonin and chromogranin A levels which indicates microbial balance affects neuroend microbiota-brain axis correlation analysis produced multiple important results. The Shannon diversity index results indicated that higherocrine parameters. The inflammatory markers demonstrated positive relationships with neuropsychological test results because CRP and IL-6 concentrations increased with higher depression and anxiety scores (Fig.1).

### Risk factors and multivariate analysis:

**Fig.2:**
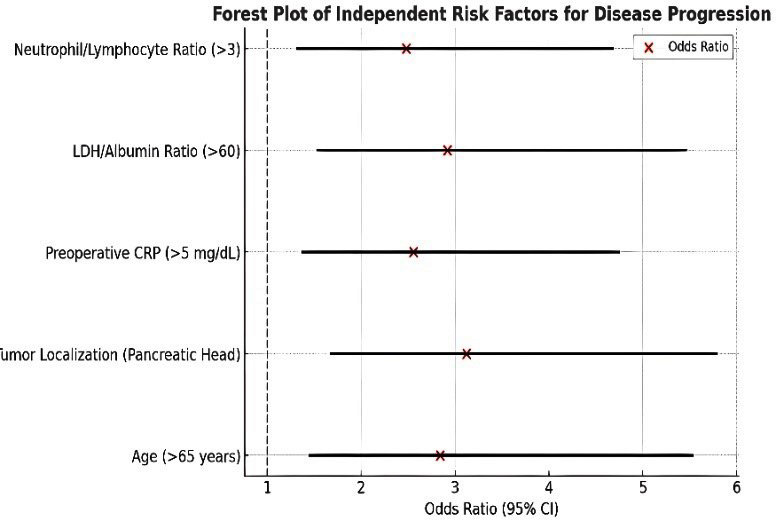
Forest Plot of Independent Risk Factors for Disease Progression. Forest plot displaying odds ratios (OR) and 95% confidence intervals for independent risk factors associated with disease progression. The vertical dashed line at OR = 1 represents the null effect. Variables with confidence intervals not crossing this line are statistically significant.

**Fig.3:**
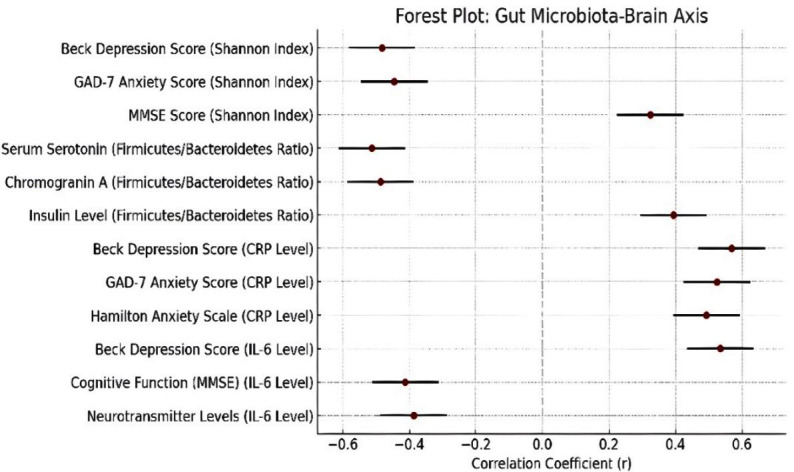
Forest Plot of Factors Influencing Gut Microbiota-Brain Axis Relationship. Forest plot showing beta coefficients (β) and 95% confidence intervals from regression analysis of factors affecting the gut microbiota-brain axis relationship. Positive effects (blue markers) extend rightward, negative effects (red markers) leftward from the null effect line (β = 0).

The research used multivariate analysis to determine which factors independently led to disease progression. The risk factors for disease progression included patients older than 65 years and tumors located in the pancreatic head and elevated preoperative CRP levels above 5 mg/dL and high LDH/Albumin ratio values above 60 and neutrophil/lymphocyte ratio values exceeding three. The analysis of factors affecting microbiota-brain axis relationships through regression methods showed that CRP and IL-6 and TNF-α inflammatory markers and tumor size and disease stage and hospital stay duration had positive effects. The levels of albumin and hemoglobin in the blood showed negative effects on the relationship between microbiota and brain function (Fig.2 and 3).

## DISCUSSION

Our objective was to explore the involvement of the microbiota-brain axis pertaining to cystic lesions that develop in the pancreas and its possible outcomes. A specific focus was placed on assessing the microbiota composition, markers of systemic inflammation, and the neuropsychological features of the disease process in the members of the serous cystadenoma and mucinous neoplasm groups. There were notable drops in diversity of the microbiota, increases in inflammatory markers, and worsened depression-anxiety scores in patients diagnosed with mucinous neoplasm, while patients with serous cystadenoma had a higher clinical balance and more favorable microbiota composition. This evidence points towards the possibility of considering the microbiota-brain axis in the treatment of patients suffering from pancreatic cystic lesions.

We also observed that it was observed that microbiota diversity (measured by Shannon and Chao1 indices) was higher in serous cystadenoma patients compared to the mucinous neoplasm group. This finding supports the view that high microbiota diversity may have a protective role as stated by Zhang et al. in their study in 2024.^11^ The high Firmicutes/Bacteroidetes ratio found in mucinous neoplasm patients is consistent with the inflammatory processes observed by Mulders et al. in NET patients in 2024.^12^ The predominant Lactobacillus and Bifidobacterium species in the serous cystadenoma group in our study may have a positive effect on the regulation of the tumour microenvironment as emphasised by Massironi et al. in 2022.^13^ On the other hand, the increased Clostridium species in the mucinous neoplasm group is similar to the increase in pathogenic bacteria observed by Gao et al. in rectal neuroendocrine tumour patients in 2025.^14^

The increase in inflammatory markers such as CRP, IL-6 and TNF-α that we observed in mucinous neoplasm patients in our study is similar to the increase in inflammatory markers detected by Al Efishat et al. in high-risk IPMN patients in 2018.^15^ As emphasised by Chen et al. in 2020, the increase in IL-1β and inflammatory markers indicates that the disease may follow a more aggressive course.^16^ The elevation in fasting glucose, insulin and HbA1c levels that we detected in the mucinous neoplasm group in our study is consistent with the metabolic changes observed by Carmicheal et al. in 2020 in pancreatic neoplasms.^17^ As Majumder et al. noted in 2019, these deteriorations in metabolic parameters suggest that patients are at increased risk for diabetes and metabolic syndrome.^18^

Research studies have proven that probiotic supplements help control gut bacteria to treat ongoing inflammatory diseases. The research by Liu et al. (2024) demonstrated that Bifidobacteria and Lactobacilli combined with budesonide treatment successfully managed intestinal microbiota patterns in patients who had chronic obstructive pulmonary disease.^8^ The researchers found that treatment led to better microbiota diversity through elevated beneficial Lactobacillus and Bifidobacterium populations and reduced harmful bacterial numbers. The dual treatment approach brought better results in both clinical measurements and patient life quality assessments. The research focused on COPD patients but the positive changes in microbiota structure and treatment results suggest probiotics could help various diseases caused by microbial imbalances.^8^

The high depression and anxiety scores observed in patients with mucinous neoplasms in our study overlap with the findings of Farzi et al. in 2018, which emphasised the association of gut microbiota disorders with psychiatric conditions.^19^ As Alpert et al. in 2021, the negative correlation we found between decreased microbiota diversity and depression and anxiety scores supports the regulatory role of gut microbiota on neurotransmitter systems.^20^ As shown by Dono et al. in 2022, alterations in the gut microbiota can lead to neuropsychiatric symptoms by affecting metabolic functionality.^21^ As Di et al. emphasized in 2019, these findings suggest that microbiota modulation via vagal nerve activity may be a novel therapeutic approach in neuroendocrine tumours.^22^

The more frequent application of Whipple operation in patients with mucinous neoplasms in our study is consistent with the findings reported by Aizpuru et al. in 2022 that pancreaticoduodenectomy is preferred in precancerous lesions.^23^ As emphasized by Barthet et al. in 2021, the high recurrence rates observed in mucinous neoplasms and the need for aggressive surgery suggest that these tumours may have a worse prognosis.^24^ As stated by Shah et al. in 2020, the high survival rates we found in serous cystadenoma patients support that these tumours have a better clinical course.^25^

The research contains multiple important aspects which make it valuable for advancing knowledge in this field. The study achieves robust molecular characterization through its use of 16S rRNA sequencing for microbiota profiling which goes beyond traditional culture-based methods. The standardized neuropsychological assessment battery allows researchers to conduct systematic evaluations of brain-gut axis connections. The single experienced surgical team performed all procedures which reduced the impact of technical variations. The research combines biochemical and hormonal and psychological data to create a complete understanding of disease mechanisms. The study provides valuable information about different pathophysiological processes through its comparison of two distinct pancreatic cyst types.

The research results require additional studies to confirm their findings in different population groups. Research studies that follow participants over time will help scientists understand how changes in microbiota affect neuropsychological functions. Research studies that use probiotics and dietary changes as interventions should test their therapeutic value. Research into particular microbial metabolites and their neuroactive properties will help scientists understand the biological mechanisms at work. The combination of advanced imaging methods with microbiome analysis would help researchers understand how pancreatic disease affects the gut-brain connection through anatomical changes.

### Strengths of the study:

However, the strengths of our study include performing microbiota analyses with standardized protocols, using comprehensive neuropsychological assessments, and collecting detailed clinical data. In future studies, multicenter studies with larger patient populations should be planned, long-term follow-up data should be obtained, and the therapeutic efficacy of microbiota modulation should be evaluated. There is a need for randomised controlled trials investigating the effects of probiotic treatments on pancreatic cysts with neuroendocrine differentiation.

### Limitations:

It includes single center and relatively short follow-up period limit the generalisability of our results. The study lacks systematic evaluation of essential confounding elements which affect microbiota composition through dietary patterns and medication use including antibiotics and proton pump inhibitors and initial psychological state at diagnosis time. The study design prevents researchers from tracking how microbiota changes over time. The observational study design prevents researchers from determining if microbiota changes lead to disease development or if they occur after disease onset or by chance.

## CONCLUSION

This research showed how the microbiota-brain axis plays a significant role in pancreatic cysts. Our study showed that patients diagnosed with serous cystadenoma as well as those diagnosed with mucinous neoplasm exhibited differences in their microbiota composition, inflammatory responses and even their neuropsychological profiles. Microbiota imbalance, increased inflammation and high depression-anxiety levels observed especially in mucinous neoplasm patients indicate the need for a more comprehensive treatment approach in this patient group. Based on these results, it is relevant to assess the gut microbiota and the neuropsychological condition in the context of pancreatic cystic lesions diagnosis and treatment. In the future, modulation of microbiota may serve as an effective therapeutic target which may enhance the clinical outcomes of these patients.

### Author Contributions:

**IHO**: Formal analysis, Project administration, Software, Visualization and Writing - original draft. Takes full responsibility for the integrity of the work as a whole and the accuracy of the data analysis.

**IHO, OB, AO, HB:** Writing - review & editing and Investigation.

**IHO, OB:** Conceptualization, Methodology and Supervision.

**OB, AO, HB:** Data curation and Validation. Critical Review.

All authors have read and approved the final manuscript.
